# Effect of Temperature and Water Addition Ratio on the Aroma Release of Yeast Proteins

**DOI:** 10.3390/foods14061037

**Published:** 2025-03-18

**Authors:** Jiahui Chen, Dandan Pu, Boya Cao, Baoguo Sun, Yuyu Zhang

**Affiliations:** 1 Food Laboratory of Zhongyuan, Beijing Technology and Business University, Beijing 100048, China; cjh16636184361@163.com (J.C.); 18518351472@163.com (D.P.); caoboyas@163.com (B.C.); sunbg@btbu.edu.cn (B.S.); 2 Key Laboratory of Geriatric Nutrition and Health, Beijing Technology and Business University, Ministry of Education, Beijing 100048, China; 3 Key Laboratory of Flavor Science of China General Chamber of Commerce, Beijing Technology and Business University, Beijing 100048, China

**Keywords:** yeast proteins, electronic nose, aroma release, gas chromatography–mass spectrometry, sensory evaluation

## Abstract

The unique aroma of yeast proteins (YPs) influences consumer acceptance. Temperature and water content could affect the aroma characteristics of proteins. Herein, the aroma release patterns in YPs were explored via sensory evaluation, electronic nose, and gas chromatography–mass spectrometry (GC-MS). Sensory evaluation results showed that after heating, the aroma intensity of YPs initially dropped and then increased. When the water addition ratio (YPs–water) was increased from 1 to 5, the aroma intensity of plastic-like attributes decreased, whereas the remaining aroma attributes exhibited enhanced. Electronic nose analysis results were consistent with those of the sensory evaluation. Ten volatile compounds were detected in YPs. In the water–YPs model, the concentrations of only three volatile compounds decreased with increasing temperature, while the concentrations of the remaining 22 volatile compounds increased. The results elucidated the effects of temperature and water content on the aroma changes in YPs, which could provide the reference for YPs applications in different foods.

## 1. Introduction

Yeasts are single-celled fungi that can ferment sugars. Traditionally, yeast has been used to ferment bread, beer, wine, and some ethnically fermented foods and beverages in Asia and America [1]. The addition of yeast improves the aroma of the food during fermentation [2] and produces variance aroma compounds including alcoholic (ethanol, phenylethyl alcohol, isoamyl alcohol, etc.) and ester (ethyl acetate, ethyl butyrate, ethyl caprylate, etc.) [3]. Simultaneously, yeast has a high nutritional value because yeast cells mainly comprise enzymes (convertases and lactases), nucleotides, proteins (mannoproteins), polysaccharides (glycans and mannans), and lipids (phospholipids and ergosterol).

Yeast is an important source of microbial protein. The yeast protein (YP) is produced through enzymatic hydrolysis of yeast cells to generate yeast extract (YE), followed by its subsequent separation/purification from the extract. Yeast protein contains over 70% protein and has a balanced amino acid profile similar to meat and plant proteins [4]. YPs possess excellent physical and chemical properties, favorable processing characteristics, as well as high digestibility, making them widely applicable in food production, animal feed, nutritional supplements, and related fields [5]. However, YPs smell strongly of yeast, which affects their production and utilization. At present, most researchers focus on decoding the characteristic aroma of YEs. For example, the unique aroma compounds in dry yeast powder are reported to be 1-methylnaphthalene, 1,3-dimethylnaphthalene, 2,6-2-tert-butyl-1,4-benzoquinone, geranylacetone, and acetophenone [6]. Reportedly, YEs prepared from *S. cerevisiae* TGM10, *Saccharomyces boulardii* S11, and *Kluyveromyces marxianus* TGM66 exhibit a more intense fruity aroma but less intense savory and meaty aroma [7]. In a previous study, nine odorants were detected in YEs, namely butyric acid, acetic acid, propionic acid, guaiacol acid and isovaleric acid, styrene, octanal, o-xylene, and sterols with sour, musty, fatty, astringent, rancid, medicinal, and gasoline aroma, respectively. Wang et al. (2019) [8] found that 2,4-di-tert-butylphenol and methylphenol were the most potent aromatic compounds in YEs by application of the gas chromatography–olfactometry (GC–O) combined with sensory evaluation and partial least squares regression (PLSR) analysis. In addition, the *p*-cresol and indole contributing to the phenolic and animal odors of YEs were considered as off-odorants. However, only a few studies have reported on the volatile compounds of YPs. Our previous study reported 10 volatile compounds including o-cresol, (3S)-3,7-dimethyloct-7-en-1-ol, benzyl alcohol, octanal, 2-methyl-propanoic acid, butanoic acid, 3-methyl-butanoic acid, hexanal, heptanal, and indole were identified as the key aroma components in YPs by using headspace solid phase microextraction (HS-SPME)–gas chromatography–mass spectrometry–olfactory (GC-MS/O) [9].

The heating temperature, addition amount of water and salt can influence the aroma release of YPs. Cooking temperature significantly affected the organoleptic characteristics of proteins. When different temperatures were used to cook crucian carp broth, the broth cooked at 85 °C exhibited better organoleptic characteristics and more typical volatile compounds [10]. During the drying process, the content of volatile compounds in gold pomfret increases dramatically as the temperature rises [11]. Increasing temperatures cause protein unfolding, leading to aroma compounds such as aldehydes exposing their active sites, thus enabling them to bind more tightly to proteins. However, excessively high temperatures reduce the binding capacity of these aroma compounds [12]. While the presence of proteins in food matrices suppresses aroma perception and generates unpleasant odors, studies in a pure water system demonstrate that the intensity of aroma release correlates positively with both the hydrophobicity and volatility of the aroma compounds [13]. Thus, adding a certain amount of pure water to proteins affects the volatilization of aroma compounds. Nevertheless, to the best of our knowledge, regarding the pattern of aroma release from YPs in response to different temperatures and the addition of water is a lack of depth. Investigating aroma release mechanisms in YPs could enhance the detection methods for aroma compounds in YPs, leading to a more comprehensive understanding of their aroma profile. This knowledge will further support the broader use of YPs in future food applications. This study aimed to (1) investigate the aroma change patterns of YPs affected by temperature and different water addition ratios, (2) study the aroma differences in YPs under different factors through sensory evaluation and electronic nose, and (3) analyze aroma change patterns in volatile compounds in YPs under different factors using HS–SPME-GC–MS.

## 2. Materials and Methods

### 2.1. Sample Preparation

In this experiment, YPs were provided by Angel Yeast Co., Ltd. (Yichang, China). The aroma release and aroma profile changes by heating and the addition of water were investigated. Four different levels of temperature (25 °C, 35 °C, 45 °C, and 55 °C) were investigated based on the room temperature, temperature of oral processing, optimal brewing temperature of the protein powders, and better acceptable temperature of the human mouth. A total of 1.5 g of YPs were loaded into a 20 mL glass bottle and heated in a water bath. This experiment was based on the pre-experimental conditions: heating temperature of 35 °C, water added at a ratio of 1:2, and a heating time of 1 h. The different levels of single-factor settings of temperature (25 °C, 35 °C, 45 °C, and 55 °C) and the ratio of water addition (1:1, 1:2, 1:3, 1:4, and 1:5) were examined, respectively. A total of 12 experimental samples were included in this study, which were divided into three groups (e.g., temperature-treated group of dried yeast protein, temperature-treated group of turbid yeast protein, and a group of yeast protein with different water addition ratios).

### 2.2. Chemicals

2-Methyl-3-heptanone (99%) and methanol (chromatographic grade, 99.9%) were purchased from Shanghai Macklin Co., Ltd. (Shanghai, China); alkanes (C6–C30) (chromatographic grade, 99.9%) were purchased from Sigma-Aldrich Company in the St. Louis, MA, USA. Furthermore, ultrapure water was purchased from Wahaha Group (Hangzhou) Co., Ltd. (Hangzhou, China).

### 2.3. Sensory Evaluation Analysis

The aroma profile evaluation of YPs was conducted according to our previous work with some modifications [14]. The experiment was conducted by 11 trained assessors (5 male, 6 female) from Beijing Technology and Business University. Members of the sensory evaluation team were trained according to ISO 8586:2023 [15]. First, the panelists were provided with 24 odor solutions for detection and training, identifying each odor sample and describing the odor or making an association. After giving the correct answers from each panel, they were finally selected for the final sensory evaluation section.

Samples were presented to evaluators for quantitative sensory description assessment. First, the YP samples were presented to the panelists, and the descriptors were recorded according to the statistical frequency and the sensory evaluation group discussion. According to the sensory evaluation group discussion, the final aroma descriptors were determined as sweaty, roasted, burnt, green, fermented, sour, rice bran, sweet, and plastic according to the sensory evaluation group discussion. The prepared YPs were put into a colorless, transparent, and odorless plastic bottle, randomly numbered with three digits, and presented to the sensory evaluation personnel. They were asked to rate the intensity of the aroma profiles of YPs based on a 9-point scale from 1 (very weak) to 9 (very strong). Four samples were assessed in each session, with a total of 3 independent sessions. During the evaluation process, the sensory evaluation assessors had a 5 min rest period after completion, and each sample was evaluated three times. The sensory evaluation tests were conducted according to the Helsinki Declaration. Furthermore, the sensory evaluation test was approved by the ethics committee of Beijing Technology and Business University (BTBU202333).

### 2.4. Electronic Nose Analysis

The electronic nose analysis of YPs was conducted according to our previous work with some modifications [9]. The PEN3 electronic nose (Airsense Analytics GmbH, Schwerin, Germany) came into contact with the odor emitted by the tested sample through 10 sensors, producing an instantaneous response and converting it into an electrical signal. Different sensor arrays have different response values to the odor, so as to distinguish the odor of different samples [16]. The YP sample was put in a 20 mL sample bottle and detected by electronic nose after pretreatment (heating or water addition). First, the electronic nose was cleaned at a maximum flow rate (1200 mL/min) for 1 h. Sensor parameter settings were as follows: the injection flow rate was 400 mL/min, the analysis time was 60 s, the cleaning time was 120 s, the cleaning flow rate was 400 mL/min, and the electronic nose was cleaned at a maximum flow rate of 1 h after the sample analysis. All analyses were repeated six times. The properties of the 10 sensors of the electronic nose were as follows: W1C (aromatic compounds and benzene); W5S (sensitive to nitrogen oxides); W3C (ammonia and aromatic compounds); W6S (selective mainly for hydrogen); W5C (alkanes and aromatic compounds); W1S (short-chain alkanes such as methane); W1W (sulfur organic compound); W2S (sensitive to alcohols, ethers, aldehydes and ketones); W2W (aromatic compounds and sulfur organic compound); and W3S (sensitive to long-chain alkanes) [17].

### 2.5. Aroma Extraction via HS-SPME

HS-SPME was used to extract the volatile compounds from the YP samples. A total of 1.5 g of sample and 3 mL of water were placed into a 40 mL transparent glass bottle with a magnetic stirring rotor. Furthermore, 10 μL of 2-methyl-3-heptanone (0.100 mg/mL dissolved in methanol) was added as an internal standard. The vial was sealed with a septum screw cap. Bottles containing samples were equilibrated at different temperatures for 20 min. The DVB/PDMS fibers were exposed in the bottle for extraction for 40 min. The fibers were then quickly inserted into the injection port for desorption at 250 °C for 5 min with the pulse splitless mode.

### 2.6. GC–MS

The qualitative and quantitative analysis of the aroma compounds of YPs were performed using an Agilent 8890 GC (Agilent Technologies, Santa Clara, CA, USA) equipped with a 5977B mass selective detector. The volatile compounds were separated on a DB-WAX column (30 m × 0.25 mm, inner diameter 0.25 μm). Ultrapure helium (99.999%) was used as the carrier gas at a flow rate of 1.00 mL/min. The pulse splitless mode was used during these analyses. The aroma isolation and detection parameters of GC–MS was conducted according to our previous study [9]. Oven temperature program was as follows: initial temperature was maintained at 35 °C for 1 min, then increased to 100 °C at 4 °C/min and held for 1 min, then increased to 170 °C at 2 °C/min, held for 1 min, and finally increased to 220 °C at 5 °C/min and held for 1 min. All the analyses were repeated in triplicate.

### 2.7. Qualitative and Quantitative Methods

GC–MS data were analyzed Via Qualitative Analysis 10.0 software (Agilent Technologies, Santa Clara, CA, USA), and compared with the NIST20 database. The compounds were identified based on the retention index (RI) and spectral library (MS). The RI was calculated based on a series of standard linear alkanes (C6–C30).

The concentration of each volatile compound was obtained based on the ratio of the peak area of the volatile compound to the peak area of the internal standard. The concentration of each volatile compound was calculated based on Equation (1).

(1) $$content\;(\mu g/kg)=\frac{SC_{I}}{mS_{I}}\times 10^{3}$$

where S is the peak area of volatile compounds, S_I_ is the peak area of the internal standard, C_I_ is the level of the internal standard, and m is the mass of the sample.

### 2.8. Statistical Analysis

All data were performed in triplicate and the results were expressed as mean ± standard deviation (mean ± SD). Data were statistically analyzed using SPSS Statistics 24 software with ANOVA and the Duncan test (*p* < 0.05). The figures were drawn by using Origin 2024 (Origin Lab, Northampton, MA, USA). The PCA analyses in this study were performed based on the raw data (not the mean of the replicates) from three independently replicated experiments.

## 3. Results and Discussion

### 3.1. Sensory Evaluation

**Figure 1:**
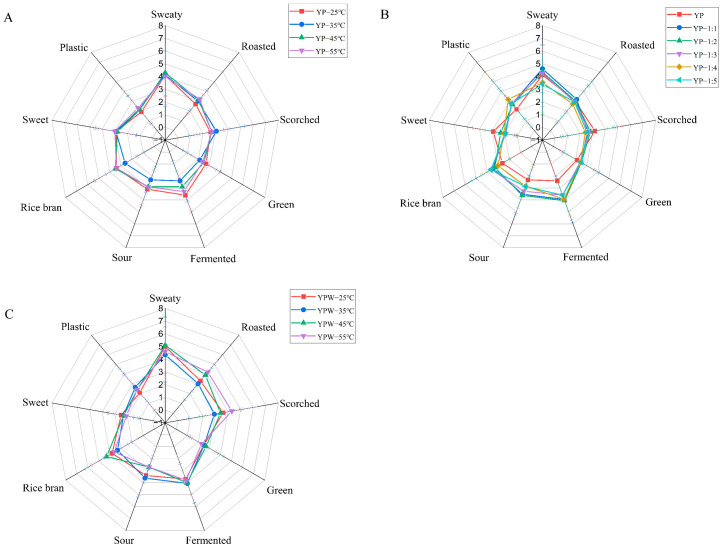
Radar chart of yeast proteins (YPs) sensory evaluation ((**A**) dry YPs at different temperatures, (**B**) different water ratios of YPs, (**C**) YP emulsion at different temperatures).

The quantitative descriptive analysis was performed to obtain the overall aroma profile of YPs at different temperatures (Figure 1). Results showed that the intensity of sweaty, fermented, and rice bran aromas of YPs were the highest after heating at high temperatures, followed by roasted, scorched, green, and sweet notes. The difference among the YP samples was the most pronounced in sweaty aroma compared with the other YPs. The YPs heated at 35 °C had the lowest value of overall aroma intensity, so the YPs heated at 35 °C were chosen for the subsequent studies. Figure 1B shows the aroma characteristics of YPs after the reintroduction of pure water, and the intensities of sweaty, fermented, and sour differed greatly from those of the dry YPs after the addition of water. The aroma intensities of YPs after water addition were not significantly changed, but it was different from the dry YPs. Figure 1C shows the aroma characteristics of YPs after the addition of water under heating at different temperatures. The intensities of sweaty, scorched, and fermented aromas were the highest after the addition of water, followed by roasted, rice bran, and sweet notes, and the intensities of plastic and green aromas were the lowest.

### 3.2. Sensor Array Response to YP Samples

**Figure 2:**
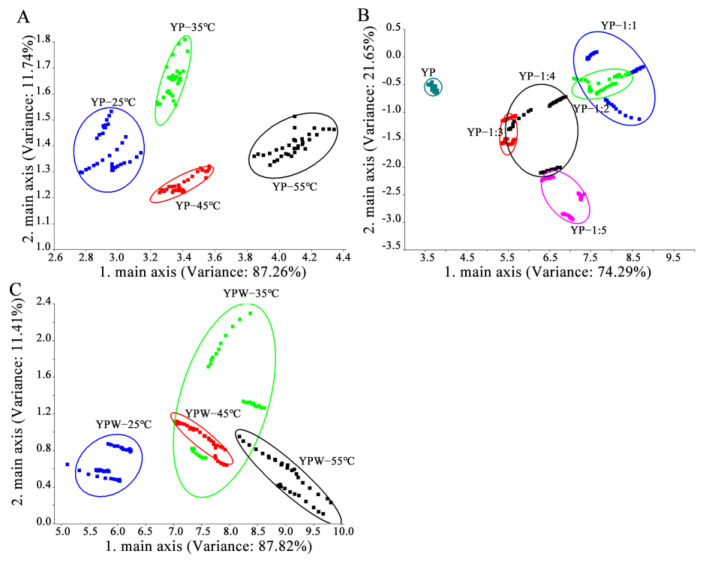
Principal component analysis plots of electronic nose data ((**A**) dry YPs at different temperatures, (**B**) different water ratios of YPs, (**C**) YP emulsion at different temperatures).

The aroma characteristics of YPs after different treatments (water content and heating) were analyzed using the electronic nose (Figure 2). Principal component analysis (PCA) of the electronic nose data was employed to determine significant differences between samples based on olfactory sensor information [18]. As shown in Figure 2A, PCA could differentiate YPs processed at different temperatures. PC1 and PC2 accounted for more than 90% of the variance (87.26% and 11.74%, respectively), indicating that they effectively captured the overall characteristics of the sample data. The YPs heated at 55 °C were located on the far right of the PCA score plot, far away from other samples, indicating that their aroma was significantly different. As shown in Figure 2B, PC1 and PC2 accounted for over 90% of the variance (74.29% and 21.65%, respectively), reflecting the overall characteristics of the sample data. However, PCA failed to distinguish samples with water addition ratios ranging from 1:1 to 1:5. Specifically, the aroma characteristics of samples with water addition amounts of 1:1 and 1:2 overlapped, and the aroma characteristics of samples with water addition amounts of 1:3, 1:4, and 1:5 overlapped. The dry powder sample was located in the lower left corner of the figure and did not intersect with the aroma contours of other samples, indicating that the aroma of the dry powder sample significantly differed from other samples. As shown in Figure 2C, PCA could not distinguish YP suspensions heated at 35 °C, 45 °C and 55 °C, indicating that the aroma of the corresponding samples did not change significantly or it might be because the aroma intensity lowered after heating. The YPs heated at 25 °C were located in the lower left corner of the picture, indicating that the aroma of YPs heated at 25 °C differed substantially from the aroma of YPs heated at 35 °C, 45 °C, and 55 °C.

### 3.3. Changes in Volatile Compounds of YPs at Different Temperatures

**Figure 3:**
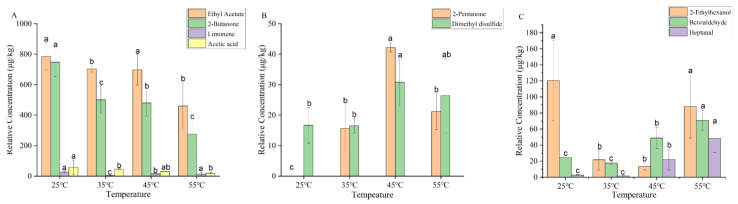
Changes in the concentrations of volatile compounds in dry YPs at different heating temperatures ((**A**) changes in the concentrations of ethyl acetate, 2-butanone, limonene, and acetic acid in dry YPs at different heating temperatures; (**B**) changes in the concentrations of 2-pentanone and dimethyl disulfide in dry YPs at different heating temperatures; (**C**) changes in the concentrations of 3-methyl-2-butanone, 2-ethylhexanol, benzaldehyde, and heptanal in dry YPs at different heating temperatures). Letters (a, b, c) indicate significantly difference at *p* < 0.05 (Duncan test).

A total of 10 volatile aroma compounds, including alcohols, aldehydes, ketones, esters, acids, hydrocarbons, and others were detected in the dry YPs by using GC–MS combined with DB-WAX column (Figure 3). The concentrations of ethyl acetate, 2-butanone, 3-methyl-2-butanone, and 2-ethylhexanol decreased (*p* < 0.05) in dry YPs with increasing temperature, indicating that high temperature reduced the release of ethyl acetate and 2-butanone in dry yeast powder. Ethyl acetate provides pear and banana-like aromas in the dry YPs [19]. 2-Butanone was produced by the thermal degradation of fatty acids. Lower temperatures were found to slow down this process [20]. 3-Methyl-2-butanone was produced by the Maillard reaction during thermal processing [21]. The process of heating at low temperatures was found to inhibit the occurrence of the Maillard reaction. 2-Ethylhexanol was produced by the oxidative degradation of triglycerides, with orange and fatty aromas [22]. Temperatures of 35 °C and 45 °C inhibit the oxidative degradation of triglycerides, contributing to the reduction of 2-ethylhexanol. A temperature of 55 °C promotes the oxidation reaction, thereby increasing the concentration of 2-ethylhexanol. The concentrations of heptanal and benzaldehyde rise with increasing heating temperature. Previous studies [23] have demonstrated that heating induced the reduction in both the overall surface hydrophobicity and the exposure of sulfhydryl groups in proteins. This result led to a reduction in the binding of heptanal to the proteins, resulting in the release of more free heptanal into the air.

### 3.4. Effects of the Water Addition Ratio on Volatile Compound Level of YPs at the Same Temperature

Selected aroma compounds in YPs were quantitatively analyzed with different water addition amounts using the SPME method (Table 1). A total of 8 volatile compounds were detected in the dry YPs, and 26, 36, 28, 33, and 32 volatile compounds were detected in the YPs with different water addition ratios of 1:1, 1:2, 1:3, 1:4, and 1:5, respectively. The addition of pure water facilitated the volatilization of the aroma compounds from YPs. However, the effect of added water content (YPs–water from 1:1 to 1:4) on the types of aroma substances in YPs was not significantly (*p* > 0.05) changed. A marked increase in compound concentration was observed when the ratio of YP-to-water was adjusted from 1:4 to 1:5 (*p* < 0.05). Notably, the concentration of volatile compounds increased with the percentage of water added, especially ethyl acetate, 2-butanone, 2-pentanone, dimethyl disulfide, hexanal, 2-heptanone, limonene, 1-hexanol, 2-nonanone, 1-nonanal, acetic acid, and benzaldehyde, suggesting that the aroma release from YPs could be facilitated by water.

### 3.5. Changes in Volatile Compounds of Aqueous YPs at Different Temperatures

**Figure 4:**
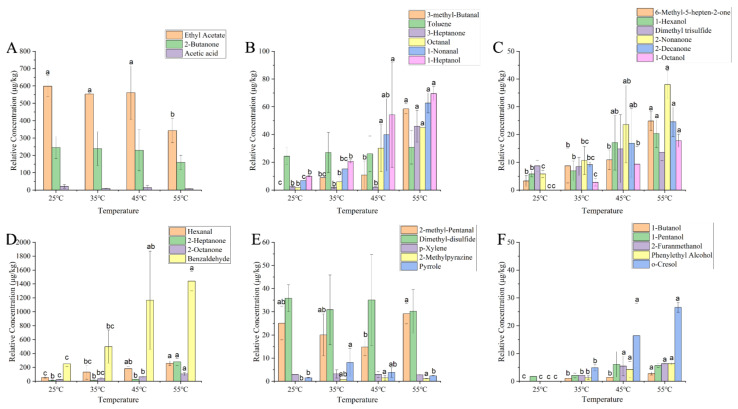
Changes in the concentrations of volatile compounds in aqueous YPs at different heating temperatures ((**A**) changes in the concentrations of ethyl acetate, 2-butanone, and acetic acid in aqueous YPs at different heating temperatures; (**B**) changes in the concentrations of 3-methyl-butanal, toluene, 3-heptanone, octanal, 1-nonanal, and 1-heptanol in aqueous YPs at different heating temperatures; (**C**) changes in the concentrations of 6-methyl-5-hepten-2-one, 1-hexanol, dimethyl trisulfide, 2-nonanone, 2-decanone, and 1-octanol in aqueous YPs at different heating temperatures; (**D**) changes in the concentrations of hexanal, 2-heptanone, 2-octanone, and benzaldehyde in aqueous YPs at different heating temperatures; (**E**) changes in the concentrations of 2-methyl-pentanal, dimethyl disulfide, *p*-xylene, 2-methylpyrazine, and pyrrole in aqueous YPs at different heating temperatures; (**F**) changes in the concentrations of 1-butanol, 1-pentanol, 2-furanmethanol, phenylethyl alcohol, and o-cresol in aqueous YPs at different heating temperatures). Letters (a, b, c) indicate significantly difference at *p* < 0.05 (Duncan test).

A total of 30 aroma compounds, including alcohols, aldehydes, ketones, esters, acids, hydrocarbons, etc., were detected in YPs after water addition using GC-MS coupled with a DB-WAX column (Figure 4). The content of ethyl acetate decreased significantly (*p* < 0.05) when the temperature was heated up to 55 °C, while the concentration of aroma compounds were not significantly changed at other temperatures levels.

The concentrations of 22 volatile compounds increased with increasing temperature. Lipid or protein oxidation usually yields aldehydes [24]. During the heating processing, the concentrations of aldehydes such as 3-methylbutyraldehyde, octanal, 1-nonanal, hexanal, and benzaldehyde increased (*p* < 0.05) with increasing temperature. 3-Methylbutyraldehyde exhibited malty and chocolatey characteristics formed by the Strecker degradation of amino groups with reducing sugar molecules in a non-enzymatic thermal reaction [25]. During the heating process, Strecker degradation occurred in the YP emulsion, increasing the concentrations of 3-methylbutyraldehyde. Octanal bound to proteins via covalent and non-covalent interactions is released more rapidly when heating promotes bond cleavage [26]. 1-Nonanal has a green and lemony aroma and is mainly produced through the oxidation of oleic acid during the heating process, which is released by heating [27]. The increase in the concentrations of hexanal occurred because the hexanal bound to proteins was released into the YPs suspension during the heating process, forming free hexanal and volatilizing into the air [28]. Reportedly, the binding between benzaldehyde and protein was reversible, and heating could affect this binding, thereby increasing the benzaldehyde concentrations.

Ketones are produced through the oxidation of unsaturated fatty acids or the thermal degradation of amino acids and mainly exhibit a fruity and fatty aroma [29]. During heating, the concentrations of ketones such as 3-heptanone, 2-heptanone, 2-octanone, 2-nonanone, 2-decanone, and 6-methyl-5-hepten-2-one increased (*p* < 0.05) along with the increase in temperature. 2-Heptanone could bind to YPs through hydrophobic interactions. Heating reduced the retention rate of 2-heptanone, thereby increasing the 2-heptanone concentrations [30]. Likewise, heat-induced proteins also released bound 2-octanone. Notably, the binding force between 2-nonanone and proteins decreased after heating, resulting in the further aroma release of the protein [31]. However, the increase in the concentrations of 2-decanone occurred because of heating, which promotes lipid oxidation in YPs [32]. In addition, 6-methyl-5-hepten-2-one is produced by plants through lycopene cleavage.

Alcohols were produced Via lipid oxidation [33], yet their thresholds were higher and had less impact on the aroma of food. During heating, the concentrations of alcohols such as 1-heptanol, 1-octanol, 2-furanmethanol, phenylethyl alcohol, and 6-methyl-5-hepten-2-one increased (*p* < 0.05) with increasing temperature. 1-Heptanol can combine by hydrogen bonds, van der Waals force, and static and hydrophobic interactions with proteins in a reversible manner. As the temperature rises, molecular interactions intensify [34]. The increase in the concentrations of 1-octanol may have occurred because of the accelerated oxidation of oleic acid in YPs during heating [35]. 1-Pentanol is the oxidation by-product of linoleic acid. Heating can promote oxidation and accelerate the production pathway of 1-pentanol, but higher temperatures may be required [36]. 2-Furanmethanol is afforded from the interaction of fatty aldehydes and the Maillard reaction, which can be promoted through heating [37]. Furthermore, YPs contain a large amount of phenylalanine, which promotes the biosynthesis of phenylethyl alcohol.

The concentrations of o-cresol gradually increased (*p* < 0.05) which was consistent with the sensory evaluation results that the intensity of plastic aroma increased. The concentrations of dimethyl disulfide and dimethyl trisulfide did not change as it was dependent on sulfur-containing compounds, such as methionine in YPs, which were produced through protein oxidation, resulting in the concentrations of methionine not changing during heating [24].

## 4. Conclusions

The effects of heating temperature and water addition ratio on the aroma release of YPs were investigated. The sensory evaluation results showed that the sweaty and fermented aroma of YPs exhibited the highest intensity. Additionally, the aroma profile of aqueous YPs significantly differed from that of the dry YPs. Electronic nose results revealed significant differences between the dry YPs and YPs added with water; however, no significant differences were observed in the aroma release of the YP samples after the addition of different amounts of water. Interestingly, after heating at 35 °C and 45 °C, the YP samples could not be distinguished by the electronic nose, suggesting that both the types and relative concentrations of VOCs responsible for sensor responses were not significantly different under these conditions. A total of 10 volatile compounds were detected in the dry YPs. A total of 8 volatile compounds were detected in the dry YPs, and 26, 36, 28, 33, and 32 volatile compounds were detected in the YPs with the water addition ratios of 1:1, 1:2, 1:3, 1:4, and 1:5, respectively. Thus, the addition of purified water was beneficial to the volatilization of aroma compounds in YPs. More than 30 volatile compounds were detected in YPs at different heating temperatures after water addition. Furthermore, the levels of only 3 volatile compounds decreased with increasing temperature, while those of 22 volatile compounds increased with increasing temperature, which may be due to the increasing temperature facilitating the release of volatile compounds. Simultaneously, during heating, YPs underwent degradation and oxidation of fatty acids, and new substances were generated. Future research should focus on elucidating the fundamental mechanisms underlying odor alterations in YPs. This mechanistic understanding will provide critical insights for optimizing the applications of YPs in food industrial and sustainable manufacturing systems.

## Figures and Tables

**Table 1:** Identification and quantification of aroma active compounds in yeast proteins (YPs) with different ratio of water by SPME-GC-MS.

No.	Compounds	CAS Number	RI ^#^	Relative Content (mg/L)					
				YPs	YPs: Water (1:1)	YPs: Water (1:2)	YPs: Water (1:3)	YPs: Water (1:4)	YPs: Water (1:5)
1	Butanal	123-72-8	866/876	ND	ND	4.57 ± 1.50 ^b^	ND	ND	18.11 ± 0.00 ^a^
2	Ethyl Acetate	141-78-6	921/886	527.54 ± 20.67 ^d^	1758.90 ± 200.00 ^bc^	394.87 ± 224.95 ^d^	2008.29 ± 12.55 ^b^	1585.16 ± 366.55 ^c^	2790.76 ± 105.13 ^a^
3	2-Butanone	78-93-3	908/897	ND	915.51 ± 20.10 ^a^	238.51 ± 97.50 ^a^	1173.15 ± 37.39 ^a^	778.27 ± 200.63 ^a^	1167.30 ± 128.22 ^a^
4	3-Methyl-butanal	590-86-3	905/903	ND	13.16 ± 0.00 ^b^	8.95 ± 0.00 ^c^	ND	4.39 ± 2.20 ^d^	15.51 ± 2.14 ^a^
5	3-Methyl-2-butanone	563-80-4	925/960	ND	9.80 ± 3.70 ^b^	55.30 ± 20.24 ^a^	ND	ND	ND
6	2-Pentanone	107-87-9	990/965	19.80 ± 4.84 ^c^	134.81 ± 61.09 ^b^	ND	132.92 ± 60.64 ^b^	194.13 ± 47.68 ^b^	318.13 ± 20.39 ^a^
7	Pentanal	110-62-3	982/987	ND	ND	0.68 ± 0.37	ND	ND	ND
8	Toluene	108-88-3	1042/1021	ND	84.36 ± 36.62 ^ab^	27.11 ± 14.41 ^c^	44.76 ± 18.91 ^bc^	68.57 ± 6.55 ^b^	117.15 ± 29.84 ^a^
9	3-Hexanone	589-38-8	1058/1037	ND	7.02 ± 0.36	ND	ND	ND	ND
10	Dimethyl disulfide	624-92-0	1071/1058	16.54 ± 2.43 ^b^	53.95 ± 6.07 ^b^	30.85 ± 15.03 ^b^	56.49 ± 36.47 ^b^	91.42 ± 24.84 ^b^	233.00 ± 107.43 ^a^
11	Hexanal	66-25-1	1089/1069	ND	142.33 ± 14.32 ^b^	130.86 ± 102.00 ^b^	185.25 ± 101.92 ^b^	192.12 ± 20.24 ^b^	641.69 ± 103.71 ^a^
12	p-Xylene	106-42-3	1149/1129	ND	3.63 ± 0.88 ^b^	3.15 ± 1.80 ^b^	5.16 ± 0.42 ^b^	18.81 ± 5.73 ^a^	16.01 ± 4.69 ^a^
13	1-Butanol	71-36-3	1136/1138	ND	2.84 ± 2.14 ^a^	1.01 ± 0.68 ^b^	ND	ND	ND
14	3-Heptanone	106-35-4	1131/1144	ND	2.34 ± 0.00 ^b^	1.88 ± 0.50 ^b^	2.61 ± 0.00 ^b^	5.86 ± 3.55 ^a^	ND
15	2-Heptanone	110-43-0	1191/1174	ND	46.33 ± 26.17 ^b^	15.93 ± 2.84 ^b^	26.94 ± 9.99 ^b^	56.47 ± 22.53 ^b^	174.16 ± 70.50 ^a^
16	Heptanal	111-71-7	1174/1178	1.64 ± 0.24 ^c^	117.41 ± 51.93 ^a^	33.85 ± 20.30 ^c^	60.10 ± 39.35 ^abc^	46.57 ± 13.90 ^bc^	105.34 ± 58.42 ^ab^
17	Limonene	138-86-3	1195/1190	4.50 ± 2.65 ^d^	38.42 ± 27.90 ^cd^	7.04 ± 5.24 ^d^	86.21 ± 32.39 ^bc^	108.51 ± 54.33 ^b^	181.50 ± 35.61 ^a^
18	1-Pentanol	71-41-0	1249/1246	ND	10.16 ± 1.69 ^c^	2.10 ± 0.83 ^c^	7.23 ± 3.21 ^c^	17.85 ± 4.19 ^b^	31.14 ± 7.97 ^a^
19	Styrene	100-42-5	1260/1254	ND	2.65 ± 0.00 ^c^	1.92 ± 1.46 ^cd^	1.25 ± 0.00 ^d^	25.16 ± 0.00 ^a^	7.79 ± 1.37 ^b^
20	2-Methylpyrazine	109-08-0	1265/1259	ND	4.58 ± 4.23 ^b^	0.80 ± 0.00 ^b^	1.17 ± 0.00 ^b^	2.87 ± 0.22 ^b^	8.73 ± 6.25 ^a^
21	Acetoin	513-86-0	1292/1275	ND	ND	ND	3.36 ± 0.00	ND	ND
22	2-Octanone	111-13-7	1283/1278	ND	44.24 ± 23.39 ^b^	36.13 ± 15.81 ^b^	24.77 ± 6.14 ^b^	47.71 ± 1.83 ^ab^	67.29 ± 9.20 ^a^
23	Octanal	124-13-0	1270/1287	ND	ND	5.90 ± 3.63 ^c^	ND	39.52 ± 4.03 ^b^	46.78 ± 8.17 ^a^
24	6-Methyl-5-hepten-2-one	110-93-0	1330/1329	ND	14.95 ± 8.50 ^b^	8.72 ± 6.18 ^b^	6.70 ± 1.85 ^b^	16.11 ± 0.00 ^b^	75.31 ± 17.23 ^a^
25	2,6-Dimethyl-pyrazine	108-50-9	1315/1331	ND	ND	ND	ND	9.08 ± 0.00	ND
26	1-Hexanol	111-27-3	1349/1348	ND	15.47 ± 9.75 ^c^	6.89 ± 3.60 ^c^	14.91 ± 7.96 ^c^	37.36 ± 9.44 ^b^	58.82 ± 1.47 ^a^
27	Dimethyl trisulfide	3658-80-8	1378/1374	ND	12.93 ± 4.75 ^ab^	8.47 ± 3.30 ^ab^	5.25 ± 2.98 ^ab^	18.92 ± 5.11 ^a^	26.97 ± 2.71 ^a^
28	2-Nonanone	821-55-6	1387/1383	ND	ND	10.67 ± 5.04 ^c^	13.97 ± 0.97 ^c^	32.18 ± 2.34 ^b^	39.07 ± 0.20 ^a^
29	1-Nonanal	124-19-6	1388/1390	ND	ND	15.29 ± 4.94 ^b^	16.50 ± 0.00 ^b^	69.07 ± 13.09 ^a^	92.52 ± 19.43 ^a^
30	Acetic acid	64-19-7	1451/1416	ND	20.56 ± 12.84 ^c^	8.49 ± 1.17 ^c^	71.89 ± 5.13 ^b^	89.03 ± 16.17 ^b^	160.71 ± 16.14 ^a^
31	Formyl acetate	2258-42-6	1436/1449	12.70 ± 9.06	ND	ND	ND	ND	ND
32	1-Heptanol	111-70-6	1455/1452	ND	ND	ND	102.30 ± 20.23 ^c^	136.42 ± 23.40 ^b^	160.71 ± 16.14 ^a^
33	Methyl 3-hydroxybutyrate	1487-49-6	1454/1474	ND	ND	4.91 ± 0.00	ND	ND	ND
34	2-Ethylhexanol	104-76-7	1492/1486	21.69 ± 12.83 ^a^	ND	6.67 ± 0.00 ^b^	ND	ND	ND
35	2-Decanone	693-54-9	1489/1487	ND	ND	9.13 ± 0.89 ^c^	ND	30.67 ± 4.94 ^b^	45.23 ± 4.75 ^a^
36	Pyrrole	109-97-7	1498/1505	ND	4.17 ± 1.82 ^b^	8.10 ± 6.09 ^b^	7.91 ± 3.51 ^b^	12.74 ± 1.83 ^b^	30.51 ± 14.22 ^a^
37	Benzaldehyde	100-52-7	1503/1511	17.62 ± 1.55 ^c^	874.39 ± 74.22 ^abc^	499.11 ± 238.56 ^bc^	1824.74 ± 221.36 ^ab^	2150.01 ± 328.14 ^ab^	3586.72 ± 185.60 ^a^
38	1-Octanol	111-87-5	1564/1555	ND	ND	2.82 ± 1.36 ^c^	9.37 ± 6.58 ^b^	ND	33.46 ± 3.84 ^a^
39	2-Furanmethanol	98-00-0	1640/1651	ND	ND	2.11 ± 0.00 ^c^	ND	11.78 ± 1.26 ^b^	23.12 ± 1.74 ^a^
40	1-Nonanol	143-08-8	1661/1657	ND	ND	2.76 ± 1.27 ^c^	8.01 ± 9.69 ^c^	23.25 ± 4.56 ^b^	39.04 ± 3.35 ^a^
41	Benzyl alcohol	100-51-6	1879/1862	ND	ND	ND	ND	8.65 ± 2.11 ^b^	14.59 ± 2.21 ^a^
42	Phenylethyl Alcohol	60-12-8	1873/1896	ND	ND	1.23 ± 0.69 ^c^	ND	10.38 ± 4.01 ^b^	14.59 ± 2.21 ^a^
43	o-Cresol	95-48-7	1934/1897	ND	9.46 ± 3.68 ^c^	4.85 ± 1.11 ^c^	6.48 ± 4.53 ^c^	31.36 ± 6.38 ^a^	17.81 ± 0.00 ^b^

#, retention index (RI) value is literature/calculation; ND, not detected; Letters (a, b, c, d) indicate significantly difference at *p* < 0.05 (Duncan test).

## Data Availability

The original contributions presented in this study are included in the article. Further inquiries can be directed to the corresponding author.
